# Open-source Internet of Things (IoT)-based air pollution monitoring system with protective case for tropical environments

**DOI:** 10.1016/j.ohx.2024.e00560

**Published:** 2024-07-17

**Authors:** Edwin Collado, Sallelis Calderón, Betzaida Cedeño, Olga De León, Miriam Centella, Antony García, Yessica Sáez

**Affiliations:** a Universidad Tecnológica de Panamá, Avenida Universidad Tecnológica de Panamá, Vía Puente Centenario, Campus Metropolitano Víctor Levi Sasso 0819-0728, Panama; b Centro de Estudios Multidisciplinarios en Ciencias, Ingeniería y Tecnología AIP (CEMCIT AIP), Avenida Universidad Tecnológica de Panamá, Vía Puente Centenario, Campus Metropolitano Víctor Levi Sasso 0819-0728, Panama

**Keywords:** Air pollution, Monitoring system, Polymers, Protective cases, Sensors, 3D printing

## Abstract

In recent years, the escalation of industrial activities has significantly increased natural resource pollution, with air pollution becoming a major cause of diseases affecting living organisms. To address this critical environmental challenge, this study proposes a comprehensive air pollution monitoring system utilizing advanced technological instruments based on the Internet of Things (IoT). The system’s primary objective is to provide precise, rapid, and efficient measurements, enabling detailed examinations of pollutant behaviors and facilitating data dissemination. The system includes a monitoring station equipped with sensors to measure ambient temperature, relative humidity, and concentrations of pollutants such as carbon monoxide (CO), nitrogen dioxide (NO_2_), sulfur dioxide (SO_2_), suspended particles (PM_2.5_, PM_10_), and ozone (O_3_). Additionally, it captures meteorological variables like wind speed, wind direction, and precipitation, allowing a nuanced analysis of their correlation with air pollutants. The collected data are transmitted via the Internet and visualized on a user-friendly platform accessible from any internet-enabled device. A protective case, designed with SolidWorks CAD software and fabricated using 3D printing, was validated through simulations for extreme conditions to ensures the system’s robustness in tropical climates. The cost-effective, low-energy system offers a scalable solution for monitoring air pollution, advancing understanding of pollutant behaviors, and supporting environmental management.

## Graphical abstract

**Figure:**
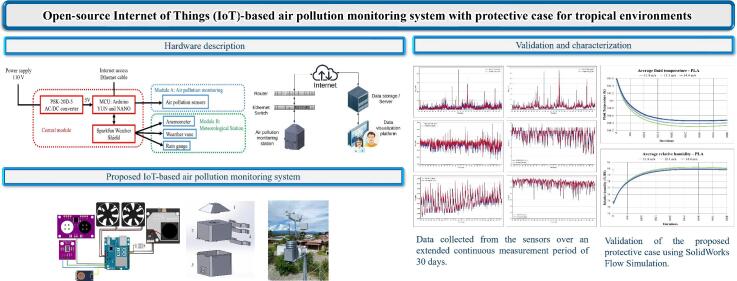

## 1 Specifications table

**Table:** 

Hardware name	Open-source air pollution monitoring system for tropical environments
Subject area	• Engineering and material science • Environmental, planetary, and agricultural sciences • Educational tools and open-source alternatives to existing infrastructure • Engineering • Internet of Things
Hardware type	• Mechanical engineering and materials science • Computer Aided Design (CAD) • Computer Fluid Dynamics (CFD) • Printed Circuit Board (PCB) • Field Measurements and Sensors • Electronics and telecommunication
Closestcommercial analog	AQM 65 Ambient Air Monitoring Station (https://www.aeroqual.com/products/aqm-stations/aqm-65-air-quality-monitoring-station)
Open-source license	https://creativecommons.org/licenses/by/4.0/
Cost of hardware	400–450 USD
Source file repository	https://doi.org/10.17605/OSF.IO/GZD62

## 2 Hardware in context

Currently, approximately 2.6 billion people are exposed to dangerous levels of air pollution, causing approximately 7 million deaths annually due to respiratory or cardiovascular diseases, which is considered the greatest environmental health risk according to the World Health Organization (WHO) [1], [2]. The main factors contributing to this problem are the rapid growth in the world population and the operation of manufacturing industries, which have considerably increased air pollution levels [3]. The International Panel on Climate Change estimates that global greenhouse gas emissions are caused by transport (14 %), energy including the generation of electricity and heat (35 %), industry (21 %), buildings (6 %), and agriculture and land use change (24 %) [4]. Air pollution not only affects the health of the population, but also has fewer known effects. Two recent studies associated pollution with unhappiness [5] and sleep problems [6], which are directly related to health or work productivity. Although measuring pollution does not automatically improve these aspects, it provides valuable information to improve the quality of life of the population.

Compared to other countries, Panama has acceptable air quality according to the study presented in Indice de calidad del aire en Panamá [7]; however, it still represents a health risk if prevention and control mechanisms are not adequately applied. According to the National Institute of Statistics and Census (INEC) of Panama, one of the major sources of air pollution is the flow of vehicles, which has increased in recent years [8]. Therefore, it is necessary to implement air pollution monitoring systems to evaluate and analyze atmospheric pollution. The process of monitoring air pollution in Panama is carried out using traditional techniques with specialized equipment placed at fixed locations to take measurements at set times. This results in an inefficient, inaccurate, and obsolete data collection. This equipment is commonly exposed to environmental agents such as rain, solar radiation, dust, temperature, and relative humidity, which affect their operation, especially in tropical climates such as in Panama, which have high levels of temperature and relative humidity for most of the year [9], [10], [11].

Recent advances in electronics and communication systems have increased the integration of Information and Communication Technology (ICT) into environmental pollution monitoring and control systems. The goal is not only to enhance the functionality and capabilities of these systems, but also to enable users to access information seamlessly from any Internet-connected device, particularly in remote locations [12], [13]. Among the most promising technologies is the Internet of Things (IoT), which comprises communication networks, sensors, actuators, and other devices to establish a fully remote monitoring and control environment [14], [15], [16]. The authors in Kumar et al. [17] propose a network intelligent gas sensor system (N-IGSS) implementing a LoRa network protocol network-link-based IoT platform, designed for deployment in restricted environments like industrial facilities and mines. The system consists of a gas sensor node with an array of seven cross-selective tin-oxide-based metal-oxide semiconductor (MOX) gas sensor elements interfaced with a low-power microcontroller and a LoRa module. Notably, it functions autonomously without reliance on Internet or GSM mobile networks. The integration of LoRa technology allows the system to be deployed in diverse applications spanning hospital management, air quality monitoring, agriculture, smart cities, and industrial operations. In Levintal et al. [18], an innovative solution for soil parameter measurement through wireless sensor networks is introduced, revolutionizing field operations by eliminating the need for sensor removal during tillage and enabling uninterrupted long-term data collection. By employing underground, wireless, open-source, and cost-effective systems to monitor soil oxygen, temperature, and moisture, this approach mitigates the complexities associated with traditional methods. In Gonzalez and Chilo [19], a web-based environmental monitoring system, leveraging Wireless Sensor Network (WSN) technology, is introduced to simplify the environmental monitoring. Comprising sensors, repeaters, and a gateway, the WSN IoT system integrates user-friendly interfaces to streamline setup complexities. The system’s efficacy is validated through testing across university and industrial environments, underscoring its adaptability and utility in diverse real-world scenarios. The work presented in Asha et al. [20], introduces an IoT-enabled Environmental Toxicology for Air Pollution Monitoring with Artificial Intelligence Technique (ETAPM-AIT), aimed at enhancing human well-being by addressing air pollution concerns. The system is composed of various sensors, data transmission via gateways, and cloud-based analytics, offering real-time assessment of air quality. The proposed idea employs the Artificial Algae Algorithm, inspired by microalgae cellular activity, for optimization, and integrates an Elman Neural Network for air quality classification. Rigorous simulations validate the effectiveness of the ETAPM-AIT model across diverse scenarios, ensuring its robust performance in safeguarding public health against hazardous pollutants. In Filho et al. [21], the proposed IoT-based monitoring system remotely measured various environmental parameters, such as air, water, atmosphere, and soil conditions, with data transmission to diverse users and remote applications. The proposed platform consists of an IoT network based on the IEEE 1451 standard which has the network capable application processor (NCAP) node (coordinator) and multiple wireless transducers interface module (WTIM) nodes. The paper further presents a dynamic data flow model that ensures network stability and efficient information exchange, offering a robust solution for long-term environmental monitoring without the risk of data loss or congestion. Another interesting work is detailed in Dhingra et al. [22], where an IoT-based three-phase air pollution monitoring system is proposed to monitor air pollution utilizing smart devices equipped with gas sensors, Arduino IDE, and Wi-Fi modules to transmit data in real-time to the cloud. A dedicated Android application, IoT-Mobair, empowers users to access and interpret air quality data, including predictive analysis for entire travel routes, akin to features in popular navigation apps. Additionally, authors in Parmar et al. [23] propose an air pollution control system that utilizes a network of low-cost air-quality monitoring nodes equipped with semiconductor gas sensors and Wi-Fi modules. The monitoring nodes measures concentrations of key air pollutants such as CO, CO2, SO2, and NO2. Data collected by the sensors is transmitted to a Raspberry Pi acting as a central base station using an ESP8266 Serial-to-Wi-Fi module that has a full TCP/UDP stack support. Data is processed and displayed in real-time on a web server powered by a MEAN (Mongo DB, Express, Angular.js, Node.js) stack.

This work proposes the design, calibration, fabrication, and validation of an open-source air pollution monitoring system for tropical environments using information technologies (ICTs), such as Internet of Things (IoT)-based networks and environmental sensors, to measure meteorological variables and concentrations of air pollutants such as carbon monoxide (CO), nitrogen dioxide (NO_2_), sulfur dioxide (SO_2_), suspended particles (PM_2.5_, PM_10_), and ozone (O_3_). This monitoring system is composed of three elements: a measuring station with sensors, a communication network, and a data-visualization platform. The operation of the monitoring system is coordinated by a central unit in charge of collecting and processing information from all connected components. The information generated by the monitoring system can be accessed by any user using an electronic device connected to the Internet. In addition to the monitoring system, this study also designed and fabricated a protective case using SolidWorks (CAD) to prevent damage caused by environmental factors such as rain, solar radiation, dust, temperature, and relative humidity. SolidWorks (CAD) provides useful tools for obtaining reliable results in thermal and fluid analyses [24]. The modeling of the housing was designed in such a way as to obtain better air flow capture and comfortable handling of the monitoring station. Each part designed in SolidWorks (CAD) was then printed on an Original Prusa 3D printer using Polylactic Acid (PLA) 3D printing filament as the manufacturing material, a resistant version for outdoor environments.

## 3 Hardware description

### 3.1 Proposed IoT-based air pollution monitoring system

**Fig.1:**
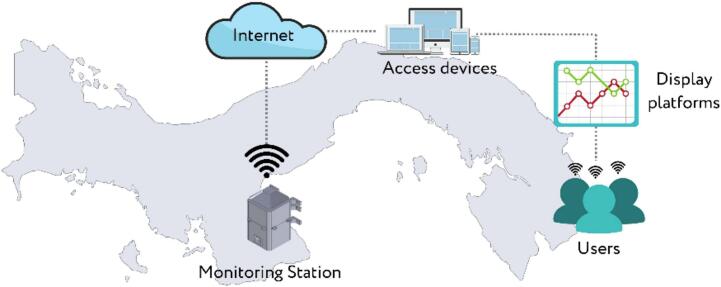
Proposed IoT-based air pollution monitoring system.

The proposed monitoring system offers a technological solution enabling users to access information concerning air pollution levels and environmental indicators. This station, located at a public university in the dry arc region of Panama, is equipped with high-performance sensors for measuring air pollutant concentrations. The location at a university also facilitates educational and research opportunities, allowing students and faculty to engage directly with the technology and data collected. Connected to the Internet via an Ethernet connection using the HTTP protocol, the station transmits real-time data to a central unit with a data visualization platform. This method was selected because of its robustness and widespread support, which enables seamless integration with existing network infrastructures and easy accessibility for users across the university. From any Internet-connected device, users can access, visualize, and analyze the data through tabular or graphical representations. Fig. 1 shows the schematic of the proposed IoT-based air pollution monitoring system and the general connections between the monitoring station, the communication network, and the data visualization platform.

### 3.2 Electrical and electronic components

The critical task involved in the design of the monitoring station is the selection of the electrical and electronic components essential for system operation. Table 1 summarizes the main components of the proposed air pollution monitoring system.

**Table 1:** Main components of the proposed air pollution monitoring system.

Component	Type of component	Reference
PSK-20D-5	Power supply/module	[25]
Arduino YUN	Microcontroller	[26]
Arduino Nano	Microcontroller	[26]
MQ131	High and low concentration ozone sensor	[27], [28], [29]
MQ-136	Sulfur dioxide (SO_2_) sensor	[28], [29]
Nova SDS011 PM sensor	Particle matter sensor	[30]
MICS-6814	Carbon Monoxide (CO) and Nitrogen Dioxide (NO_2_) sensor	[31]
DHT-22	Temperature and relative humidity sensor	[32]
Meteorological Station SEN-15901 and SparkFun Weather Shield	Meteorological sensors	[33]

In this phase, it was essential to choose sensors capable of measuring the targeted pollutants, along with electrical-electronic components like the power supply and microcontrollers necessary for the system’s operation. Given that the project involves numerous sensors, ensuring the system’s optimal functionality required sufficient capacity. Consequently, we employed two microcontrollers to accommodate the diverse sensor array effectively. Table 1 summarizes the main components of the proposed air pollution monitoring system.

#### 3.2.1 Power supply

**Fig.2:**
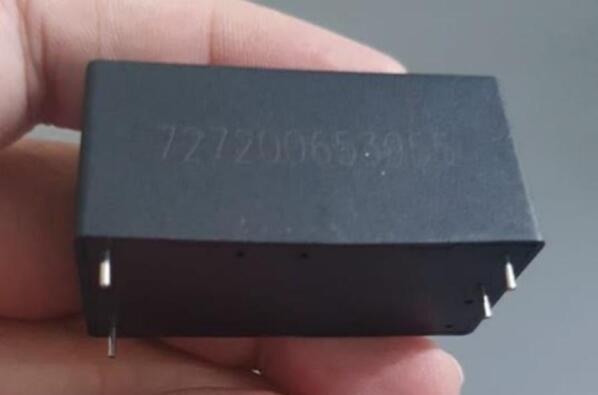
Power supply/module PSK-20D-5.

A power supply/module is a device responsible for converting the supplied voltage and current to the levels required by the system. The PSK-20D-5 used in this study supports an input of 100–277 Vac − 0.5A, 50–60 Hz, providing 5 Vdc − 4000 mA to the system. The PSK-20D-5 also supports high ambient temperatures (Ta) of 50 °C, which is necessary for adequate operation of the system [13]. Fig. 2 shows the power supply module implemented in our monitoring station.

#### 3.2.2 Single-board microcontrollers

A single-board microcontroller was built on a single-printed circuit board. This board provides all the circuitry necessary for different tasks. Given the complexity of the station, equipped with numerous sensors, two single-board microcontrollers were employed to meet the demanding requirements: Arduino YUN and Arduino Nano.

Arduino YUN

**Fig.3:**
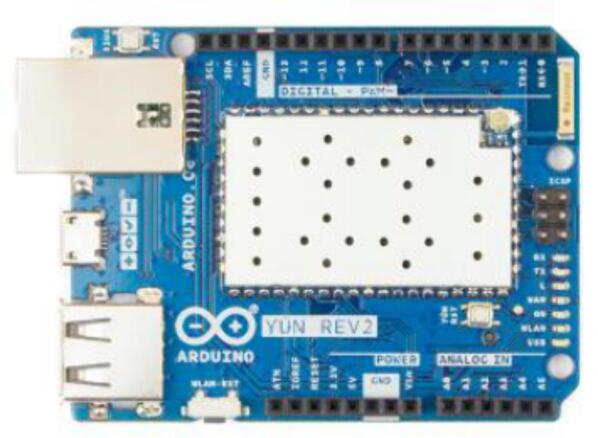
Arduino YUN REV2.

The Arduino YUN microcontroller is responsible for processing the data generated by the air pollution sensors. This device is equipped with an ATmega32U4 microcontroller and an Atheros AR9331 microprocessor running Linux, making it capable of adequately handling the tasks required at the station. The Arduino YUN also has the capacity to handle advanced network connections and applications. This device can connect to the Internet via Wi-Fi or Ethernet cables, providing flexibility for different scenarios and applications. Technical specifications are provided in Arduino [26]. Fig. 3 shows the Arduino YUN implemented in the proposed monitoring system.

Arduino Nano.

**Fig.4:**
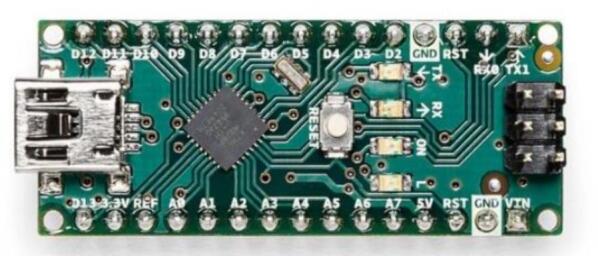
Arduino NANO.

The Arduino NANO microcontroller is responsible for processing the data generated by meteorological sensors. This device is equipped with an ATMega328 microcontroller and an analog–digital converter ADC necessary to process and convert the data generated by the analog sensors. Technical specifications are provided in Arduino [26]. Fig. 4 shows the Arduino NANO implemented in the proposed monitoring system.

#### 3.2.3 Air pollution sensors

**Fig.5:**
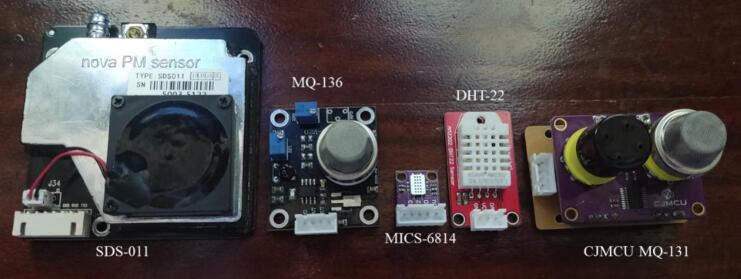
Air pollution sensors selected in the proposed system.

An importantaspect of formulating the monitoring system’s design involved meticulously choosing sensors proficient in measuring the specific air pollutants under consideration in this study. This selection process was driven by the imperative to meet dimensional requirements and ensure that the concentration ranges of each sensor align with the values and limits stipulated by the U.S. Environmental Protection Agency [34]. Fig. 5 presents the air pollution sensors used in the proposed monitoring system.

High and low concentration ozone (O_3_) sensor − CJMCU-131 MQ131[27].

The CJMCU-131 MQ131 sensor measures the high and low concentrations of ozone using Tungsten (VI) oxide as the main component. When ozone gas is present, the conductivity of the sensor decreases along with the increase in gas concentration. This sensor has high sensitivity to ozone and strong oxides such as Cl_2_ and NO_2_.

Sulfur dioxide (SO_2_) sensor − MQ-136[28].

The MQ-136 gas sensor has lower conductivity in clean air and higher conductivity in the presence of sulfur dioxide (SO_2_) along with an increase in gas concentration. This sensor has a high sensitivity to SO_2_ but can also be used to detect other sulfur-containing vapors and has low sensitivity to normal combustible gases.

Particle matter sensor − Nova SDS011 PM[30].

The Nova SDS011 PM sensor measures the concentration of suspended particles PM_2.5_ and PM_10_. This sensor uses the principle of laser scattering, which states that light scattering can be induced when particles pass through a detection area. The scattered light is transformed into electrical signals that are amplified and processed. The number and diameter of the particles can be obtained by analysis of the signal waveform, as it has certain relationships with the diameter of the particles. The Nova SDS011 PM sensor has been implemented in multiple systems because of its fast response, easy integration, high resolution (0.3 μg/m^3^), and international certification [35], [36].

Carbon Monoxide (CO) and Nitrogen Dioxide (NO_2_) sensor − MICS-6814[31].

MICS-6814 is a metal oxide semiconductor (MOS) sensor capable of detecting various gases, such as carbon monoxide, nitrogen dioxide, ethanol, methane, and propane. MOS-type gas sensors change their resistance as aresult of changes in the adsorbed concentration. MICS-6814 is a robust microelectromechanical system (MEMS) sensor for the detection of automotive exhaust pollution and agricultural/industrial odors.

#### 3.2.4 Meteorological sensors

Temperature and relative humidity sensor – DHT22[32].

**Fig.6:**
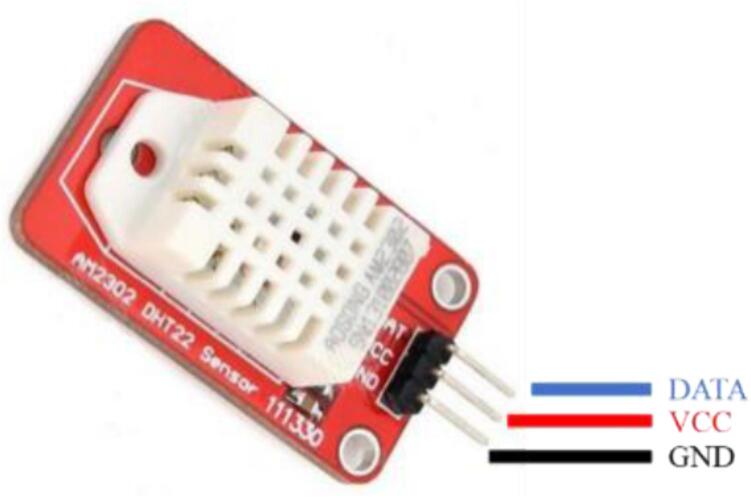
DHT22 temperature and humidity sensor.

The DHT22 temperature and humidity sensor consist of a capacitive humidity sensor and a thermistor dedicated to temperature measurement. Transmitting measurement outcomes via a digital signal is facilitated by the inclusion of a microcontroller within the sensor, tasked with executing the analog-to-digital conversion process. The temperature range is from −40 to + 80 °C, and the humidity range is from 0 to 100 %. The temperature has a precision of 0.5 °C and the humidity of 2 %. Fig. 6 shows the DHT22 temperature and humidity sensor.

Meteorological Station SEN-15901[33].

**Fig.7:**
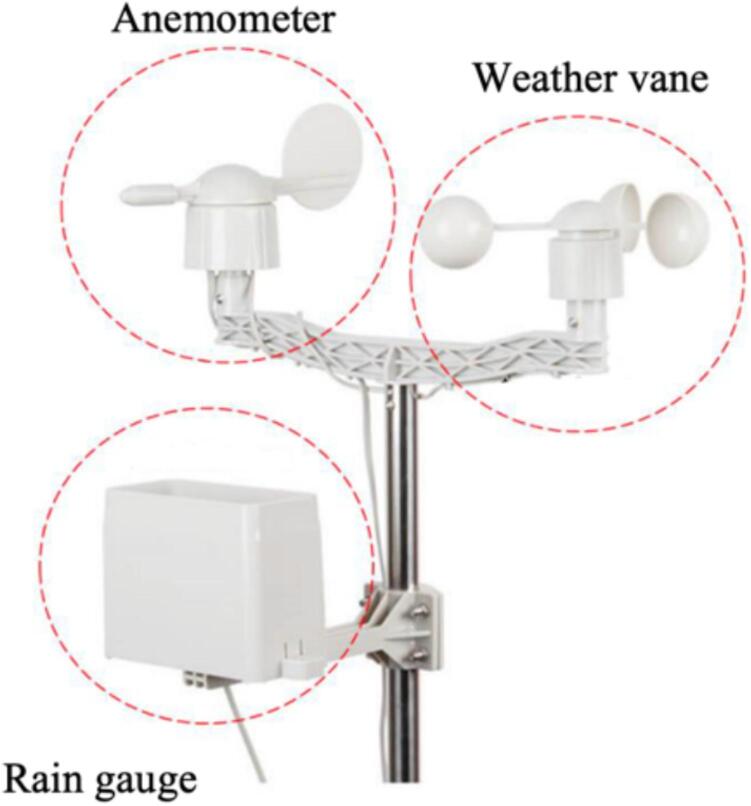
SEN-15901 Meteorological Station.

The SEN-15901 Meteorological Station consists of sensors that use magnetic reed switches and sealed magnets to generate voltages that represent the measurements. The station contains three (3) important parts: a rain gauge, an anemometer, and a weathervane. SEN-15901 uses a SparkFun Weather Shield to read weather variables and send them to the Arduino boards (Fig. 7).

Table 2 details the specifications of accuracy, precision, range, and other relevant parameters for each sensor used in our system.

**Table 2:** Specifications of air pollution and meteorological sensors.

Sensor	Detection range	Resolution	Accuracy	Precision	Operation Temperature and Humidity	Response time	Preheat time
MQ131 [27]	Low concentration: 10 ppb to 2 ppm High concentration: 0.1 to 10 ppm	10 ppb in low range 0.1 ppm in high range	±10 %	Repeatability within ±5 %	−10 °C to 50 °C and 33 % to 85 % RH	60 s	24–48 h
MQ-136 [28], [29]	1 ppm to 100 ppm	1 ppm	±10 %	Repeatability within ±5 %	−10 °C to 50 °C and 33 % to 85 % RH	<30 s	Around 24 h
Nova SDS011 PM sensor [30]	0.3 to 10 µm	0.3 µm for PM_2.5_, 1.0 µm for PM10	±10 %	Repeatability within ±5 %	−10 °C to 50 °C and 0 % to 99 % RH	<10 s	No significant preheat time required
MICS-6814 [31]	CO: 1 to 1000 ppm NH3: 1 to 500 ppm NO_2_: 0.05 to 10 ppm	CO: 1 ppm NH_3_: 1 ppm NO_2_: 0.05 ppm	±5%	Repeatability within ±5 %	−30 °C to 85 °C and 5 % to 95 % RH	<30 s	Around 48 h
DHT-22 [32]	Temperature: −40 °C to 80 °C Humidity: 0 % to 100 % RH	0.1 °C for temperature 0.1 % RH for humidity	Temperature: ±0.5 °C Humidity: ±2 % RH	High repeatability with minimal deviation	−40 °C to 80 °C and 0 % to 100 % RH	<2 s	No significant preheat time required

### 3.3 Monitoring station

**Fig.8:**
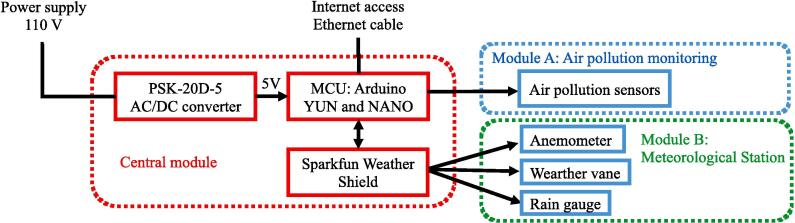
Conceptual diagram of the air pollution monitoring station.

The monitoring station is composed of a centralized modular system with one central module and two secondary monitoring modules: air pollution and meteorological variables, as shown in Fig. 8.

**Fig.9:**
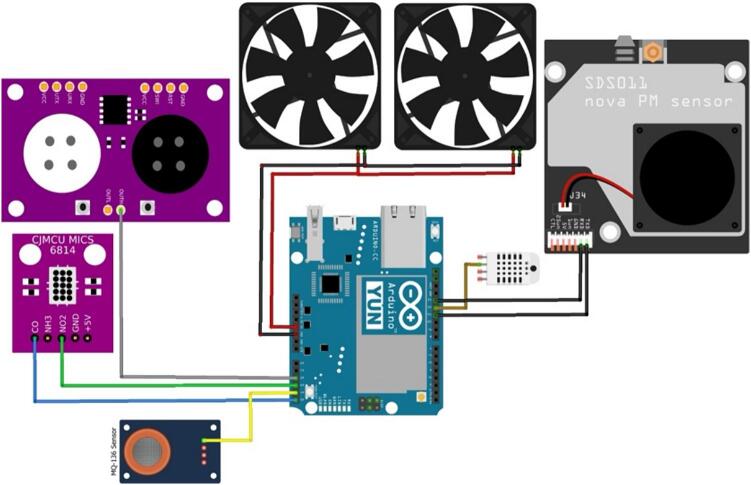
Air pollution sensor connection diagram.

In Fig. 9, the system connections involving the microcontrollers are depicted. These connections comprise the Arduino YUN microcontroller, tasked with processing data from sensors S1 to S8, representing temperature, humidity, PM_10_, PM_2.5_, CO, NO_2_, SO_2_, and O_3_, respectively. The central module governs key functions within the system, encompassing tasks such as device powering, internet connectivity, data collection and processing from sensors, supervision of sampling activities, coordination among secondary modules, and overall environmental control. Comprising essential components, the central module features a PSK-20D-5 voltage regulator responsible for converting 100–277 VAC to the 5.0 VDC necessary for powering electronic devices. It also incorporates an Arduino YUN microcontroller for air pollution monitoring, an Arduino NANO microcontroller overseeing meteorological monitoring, a SparkFun Weather Shield facilitating the connection of meteorological sensors to the Arduino NANO, and other fundamental electronic elements. Within this module, data transmission and reception are executed by the Arduino YUN microcontroller via an Ethernet connection. The air pollution monitoring module consists of four sensors measuring the concentrations of ozone (O_3_), sulfur dioxide (SO_2_), PM_2.5_, PM_10_, Carbon Monoxide (CO), and Nitrogen Dioxide (NO_2_). This module also measured the temperature and relative humidity at the station.

The meteorological monitoring module consists of the station SEN-15901, which measures the wind speed, wind direction, and precipitation. The generated information is transmitted to the Arduino NANO through the SparkFun Weather Shield, as mentioned previously.

The Build instructions section shows the design of the PCB boards for the assembly and interconnection of the electronic components of the proposed air pollution monitoring system. The designs can be seen in the repository, which were designed using EasyEDA software and printed using CNC milling machines such as CNC 3018 PRO and CNC Bantam Tools.

### 3.4 Communication network

**Fig.10:**
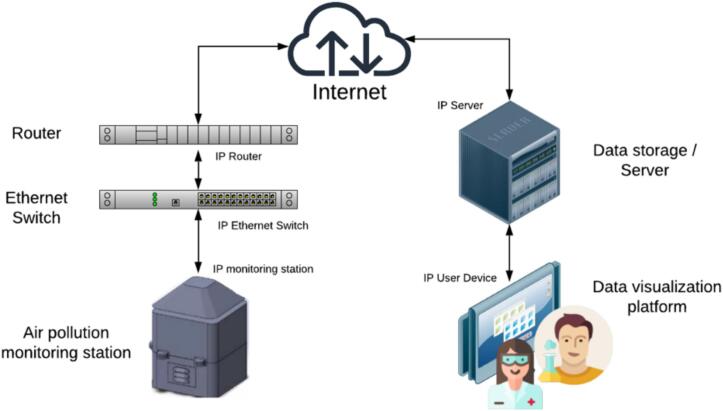
Diagram of the communication network.

The Internet assumes a major role in IoT systems, serving as the fundamental infrastructure facilitating communication, data exchange, and remote control among interconnected devices. Within this system, the monitoring station was configured to possess Internet access, allowing them to transmit sensor-generated information to the data visualization platform. This was achieved by outfitting the station with an Arduino YUN microcontroller, renowned for its capability in managing IoT applications and establishing sophisticated network connections via WiFi or Ethernet cables. Opting for Ethernet cable connectivity was preferred for its stability, faster speeds, lower latency, enhanced security, reduced interference, simplified network management, and consistent overall performance. Fig. 10 shows the diagram of the communication network.

### 3.5 Data visualization platform

Data visualization tools refer to software applications designed to present information in visual formats, such as graphs, charts, or heat maps, facilitating data analysis. These tools streamline the comprehension and manipulation of extensive datasets, enabling individuals to make data-driven decisions without investing significant time in organizing the raw data. When appropriately configured, data visualization software automates the process, extracting pertinent information from vast datasets and presenting only the most meaningful and relevant data for analysis.

The proposed air pollution monitoring system utilizes the ThingSpeak IoT platform developed by MathWorks, the company behind MATLAB. It serves as a comprehensive and user-friendly platform for collecting, analyzing, and visualizing data from connected devices. ThingSpeak simplifies the process of building IoT applications by providing a centralized space to manage and explore data streams in real-time. The key features of ThingSpeak include the ability to create and customize channels for data storage, real-time data visualization using charts and graphs, and support for various IoT devices and sensors. It also offers integration with MATLAB, allowing users to apply advanced analytics and perform complex data processing of their collected information. With ThingSpeak, users can easily connect their IoT devices to the platform, upload sensor data, and gain insight through customizable dashboards. The platform supports collaboration and sharing, enabling users to share their data publicly, or with specific individuals. In addition, ThingSpeak’s alerting capabilities allow users to set up notifications based on predefined conditions, enhancing the platform’s utility for monitoring, and responding to specific events in real-time [37].

### 3.6 Protective case

**Fig.11:**
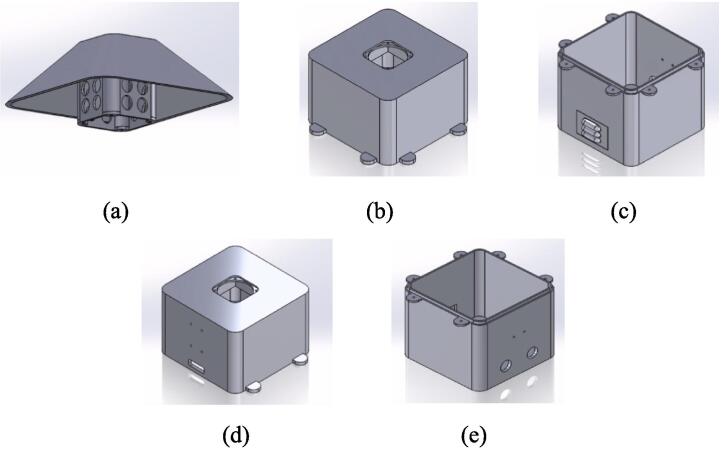
Parts of the proposed protective case: (a) Roof, (b) front view of top part, (c) front view of lower part, (d) back view of top part, (e) back view of lower part.

The proposed case was designed to protect the electrical and electronic components inside the station from environmental conditions that affect their operational conditions and cause system failure [38]. The proposed protective case comprises three parts that can be replicated or improved using Computer-Aided Design (CAD) software. The case roof in Fig. 11a is designed with holes to allow airflow and a roof-shaped plate to prevent the entry of water from rain. The top part of the case (Fig. 11b) has space to install a fan to force the entry of air at a controlled speed [39], to take sample of air quality from the outside of the station. Forced air also helps to lower the temperature inside the station, thereby improving the operational conditions of the components. The lower part (Fig. 11c) has louvers that allow the outflow of air to maintain the air circulating inside the station. According to [40], an angle of 140° in the louvers of the case low part allows the airflow to circulate freely, thereby avoiding temperature accumulation. Therefore, the louvers in the proposed case should have an inclination such that the heat can dissipate and obtain a better distribution of the fluid. Regarding their location, it is favorable to place them opposite the air inlet to allow the air mass to flow easily. In Kosutova et al. [41], it was shown that the location of the louvers plays an important role in obtaining a better air mass distribution inside the case and that the mass distribution is driven outwards. Both the top and lower parts have holes to secure the support for the case (Fig. 11d and e), and the lower part has entries to connect the cables to the energy and communication components (Fig. 11e). The top and lower parts compose the body of the protective case.

The users can modify the original design according to their requirements. This work also includes the design of accessories, such as supports for attaching cases to walls. All parts are presented in the STL format and can be printed using different 3-D printing technologies. Four 3/8 screws were utilized for assembling the case, while four 5/8 screws were employed to adjust the supports to the case.

## 4 Design files summary

This section describes the files necessary for the construction of the case. Table 3 indicates the type and location of the file.

**Fig.12:**
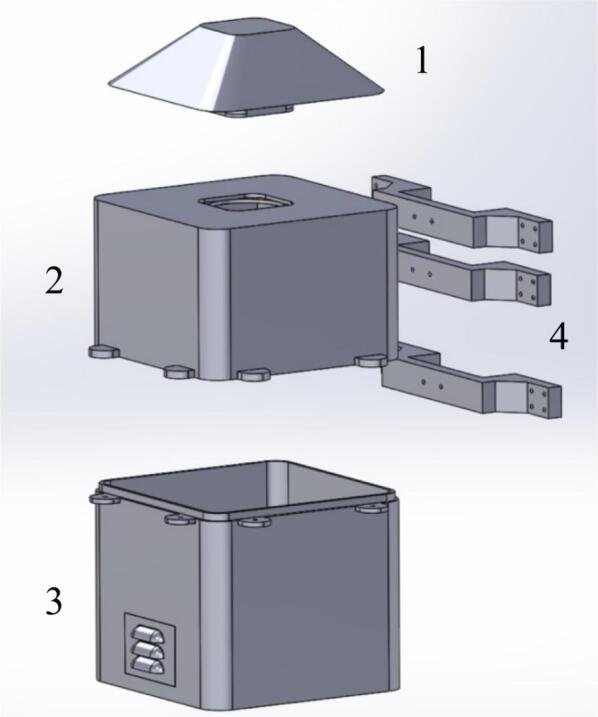
Assembled case: (1) Roof, (2) top part, (3) lower part, (4) supporters.
• The top part (part 2 in Fig. 12) represents the top part of the case. There is space for fans and notches to assemble this part with the lower part using screws.
• The lower part (part 3 in Fig. 12) represents the lower part of the case. It has louvers that allow the outflow of air, and notches to assemble this part with the top part.
• The support (part 4 in Fig. 12) was assembled to the top and lower parts of the case using screws to mount the case to any surface. Three supports are necessary to mount and secure the monitoring system on the wall.

• A plate (part 1 in Fig. 12) was used as the roof of the case, which consisted of a plate and a section with holes that allowed the entrance of air.

**Table 3:** Design files.

**Design filename**	**File type**	**Open-source license**	**Location of the file**
Plate	SLDPRT	GNU GPL v3.	https://osf.io/8c39n
TopPart	SLDPRT	GNU GPL v3.	https://osf.io/ftusc
LowPart	SLDPRT	GNU GPL v3.	https://osf.io/f59xg
Support	SLDPRT	GNU GPL v3.	https://osf.io/f9y5q
Assembly	SLDSM	GNU GPL v3.	https://osf.io/j2zfs
Simulation		GNU GPL v3.	https://osf.io/muer5
Plate	STL	GNU GPL v3.	https://osf.io/3hdgb/https://osf.io/cm5bf
TopPart	STL	GNU GPL v3.	https://osf.io/um3dy
LowPart	STL	GNU GPL v3.	https://osf.io/29dnz
Support	STL	GNU GPL v3.	https://osf.io/sm45a
Schematic	pdf	GNU GPL v3.	https://osf.io/6bp2m
PCB_complete	json	GNU GPL v3.	https://osf.io/p8euf
PCB_contamination	json	GNU GPL v3.	https://osf.io/cpq7r

## 5 Bill of materials summary

Table 4 presents the materials to be purchased for the fabrication and construction of the proposed air pollution monitoring system. It includes the cost, direct purchase link, quantity required, and type of material.

**Table 4:** Bills of materials.

**Designator**	**Component**	**Number**	**Cost per unit − cur rency**	**Total cost − currency**	**Sourceof materials**	**Material type**
PSK-20D-5	Power Supply	1	$68.91	$68.91	https://ebay.to/47QVXgB	Electronic component
Arduino YUN	Microcontroller	1	$56.40	$56.40	https://bit.ly/3TtkzqV	Electronic component
Arduino Nano	Microcontroller	1	$24.90	$24.90	https://bit.ly/3qVMnZd	Electronic component
MQ131	Ozone Sensor	1	$15.96	$15.96	https://ebay.to/3YUhKA2	Electronic component
MQ-136	SO_2_ Sensor	1	$16.13	$16.13	https://ebay.to/3qL0XCW	Electronic component
Nova PM sensor SDS011	Suspended particle Sensor	1	$5.00	$5.00	https://ebay.to/3spKl3S	Electronic component
MICS-6814	MOS Sensor	1	$20.21	$20.21	https://ebay.to/45JWqQt	Electronic component
Silver PRO Series Tough PLA Filament	PLA Filament	1	$57	$57	https://bit.ly/3pNoJx0	Polymer
4x3/8 Screws	Screws	1	$9.96	$9.96	https://bit.ly/41RkZba	Metal accessory
4x5/8 Screws	Screws	1	$13.48	$13.48	https://bit.ly/41N5mBv	Metal Accessory
IP67 waterproof and dustproof cable protector	Cable protector	2	$20.00	$20.00	https://bit.ly/3X1zCrw	Accessory
Silicone Sealant	Silicone Sealant	1	$5.15	$5.15	https://bit.ly/3oh3kvT	Glue/ Sealant
5 V DC Fan	Fan	1	$1.79	$1.79	https://bit.ly/3qJ98yV	Electronic component
9 V Battery	Battery	1	$2.01	$2.01	https://bit.ly/43Chlna	
DHT-22	Temperature and humidity sensor	1	$9.49	$9.49	https://bit.ly/3RqW9vB	Electronic component
Meteorological Station SEN-15901 and SparkFun Weather Shield	Meteorological sensors	1	$79.95	$79.95	https://bit.ly/3GIxxts	Electronic component

## 6 Build instructions

### 6.1 Printed circuit boards (PCB)

**Fig.13:**
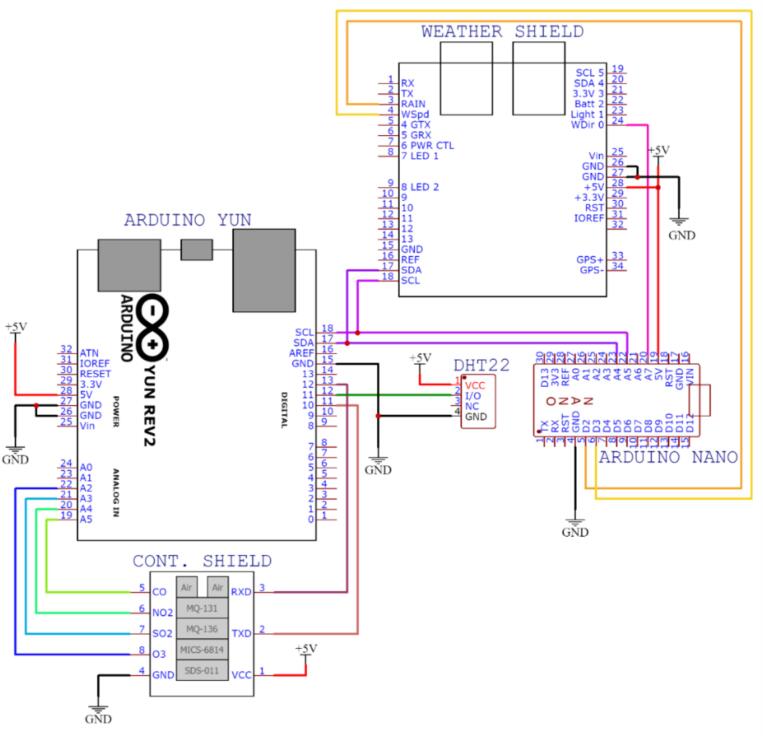
Printed circuit of the proposed air pollution monitoring station.

**Fig.14:**
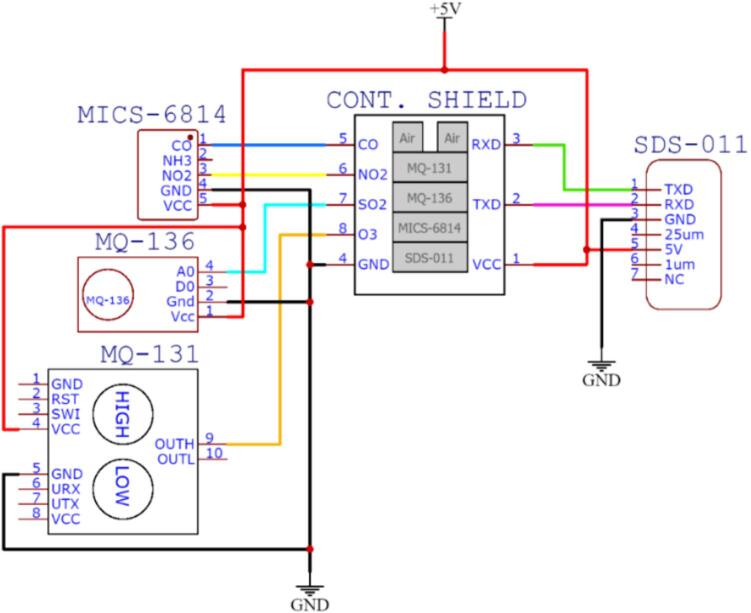
Printed circuit of the contamination shield module.

The circuit of the monitoring station was designed using the printed circuit board (PCB) design software EasyEDA, which is a cloud-based free tool that does not require installation and allows the creation of online designs. Fig. 13 shows the interconnection between the components of the proposed monitoring system, which is composed of a contamination shield module, weather shield board, air pollution sensors, meteorological sensors, and Arduino microcontrollers. Fig. 14 shows the connection between the air pollution sensors and contamination shield module.

• MICS-6814: This sensor measures carbon monoxide (blue wire) and nitrogen dioxide (yellow wire). This information is transmitted via analog pins to the contamination shield.
• MQ-136: This sensor measures sulfur dioxide (light-blue wire). This information is transmitted via analog pins to the contamination shield.
• MQ-131: This sensor measures ozone (orange wire). This information is transmitted via analog pins to the contamination shield.
• SDS-011: This sensor measures PM_2.5_ and PM_10_ particle matter. This information is transmitted to the contamination shield using serial communication from the TXD pin of the sensor to the RXD of the contamination shield (green wire), and from the RXD pin of the sensor to the TXD pin of the Contamination Shield (Cont. Shield − purple wire).

The proposed air pollution monitoring system is composed of three (3) printed circuit boards that correspond to the central module, the contamination shield module, and an adapter to facilitate the connection to the CJMCU-MQ131 sensor.

**Fig.15:**
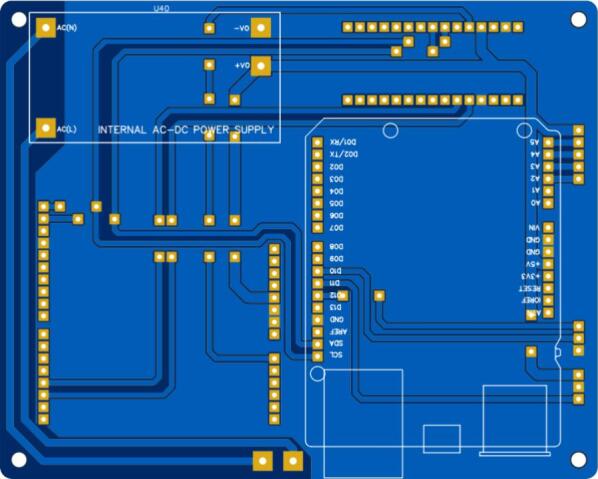
PCB for the central module of the air pollution monitoring station.

The design of the printed circuit boards for the central module of the air pollution monitoring station is shown in Fig. 15.

**Fig.16:**
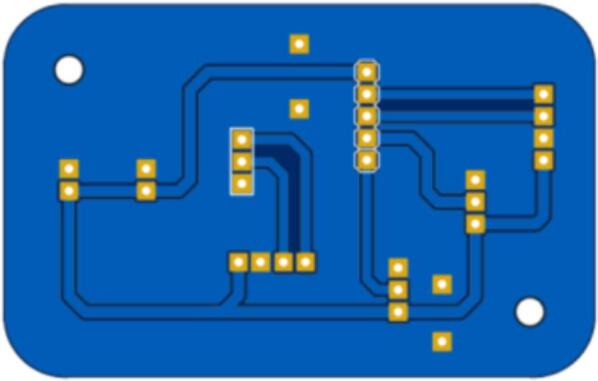
PCB for the contamination shield module of the air pollution monitoring station.

The design of the printed circuit boards for the contamination shield module of the air pollution monitoring station is presented in Fig. 16.

**Fig.17:**
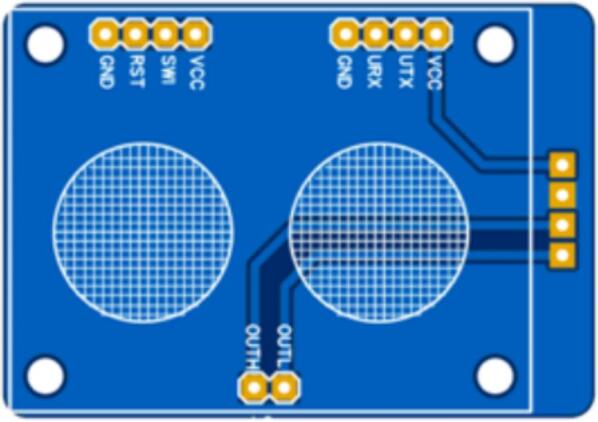
PCB for the CJMCU-MQ131 sensor adapter of the air pollution monitoring station.

The design of the printed circuit boards for the adapter to facilitate the connection to the CJMCU-MQ131 sensor of the air pollution monitoring station is presented in Fig. 17.

A 3018 PRO CNC milling machine was used to fabricate the PCBs. The process of fabricating a PCB on this milling machine may vary depending on the software used throughout the development, but basically it can be divided into three (3) parts: creation of the Gerber file, creation of.nc extension files, and printing of the PCB.

**Fig.18:**
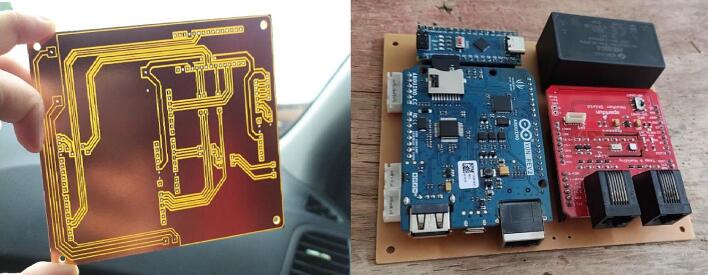
PCB for the central module.

**Fig.19:**
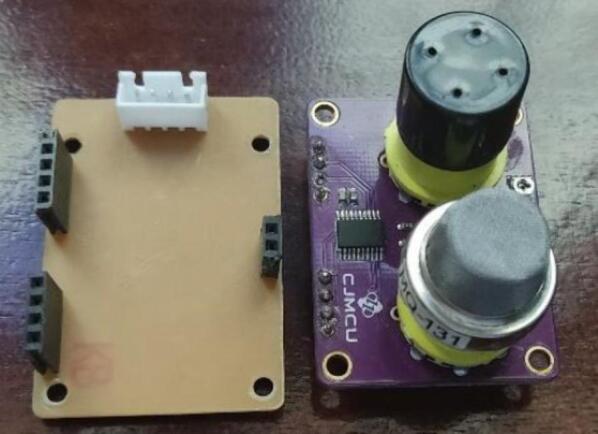
PCB for the CJMCU-MQ131 sensor adapter.

Fig. 18 shows the PCB for the central module with microcontrollers, voltage adapters, JST connectors, cables, and other electronic components considered for this station. The air pollution sensors were connected to the PCB for the contamination shield module using JST connectors. Fig. 19 shows the PCB of the CJMCU-MQ131 sensor adapter.

### 6.2 Protective case

**Fig.20:**
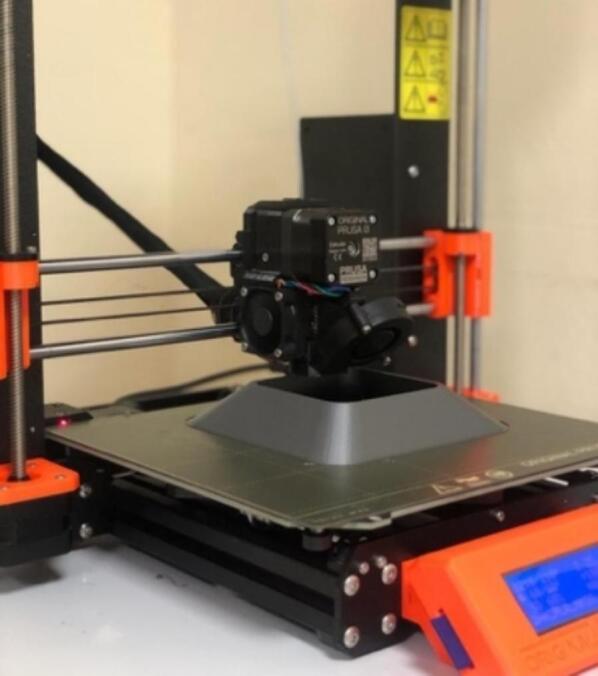
Printing process.
• The fan is inserted in the square space at the top of the protective case, as shown in Fig. 21.

The parts of the protective case shown in Fig.11, Fig.12 were printed on an Original Prusa 3D printer using as fabrication material a 3D printing filament of Polylactic Acid (PLA) – Silver PRO Series Tough PLA Filament for outdoor environments. Other accessories such as screws, cables, and cable protectors were purchased from a typical hardware store. A step-by-step guide on how to print and assemble the proposed protective case using an Original Prusa 3D printer is as follows:

• The parts are printed separately on the Original Prusa 3D printer. See Fig. 20.

**Fig.21:**
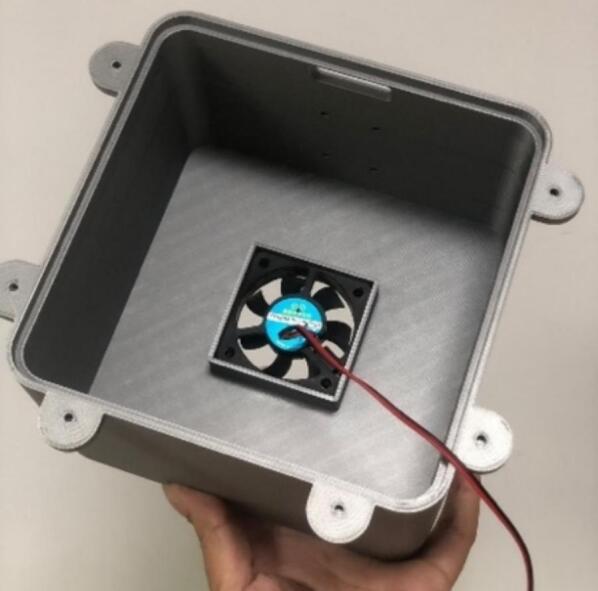
Fan colocation.
• The plate is inserted in the groove on top of the protective case. See Fig. 22.

**Fig.22:**
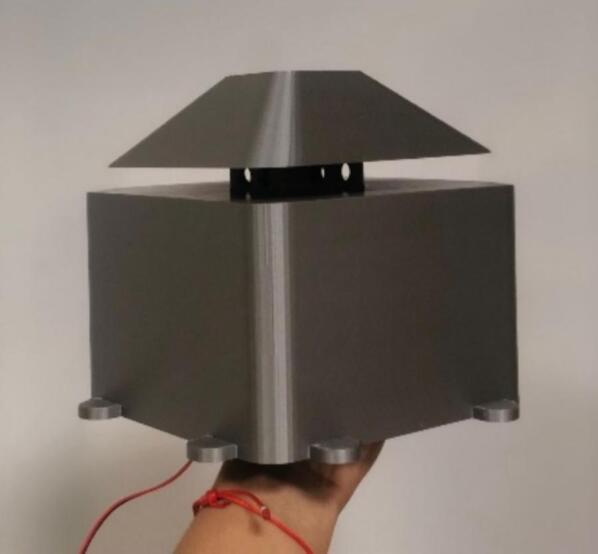
Plate on the top part.
• The fan and plate are secured by using 4 × 5/8 screws. See Fig. 23.

**Fig.23:**
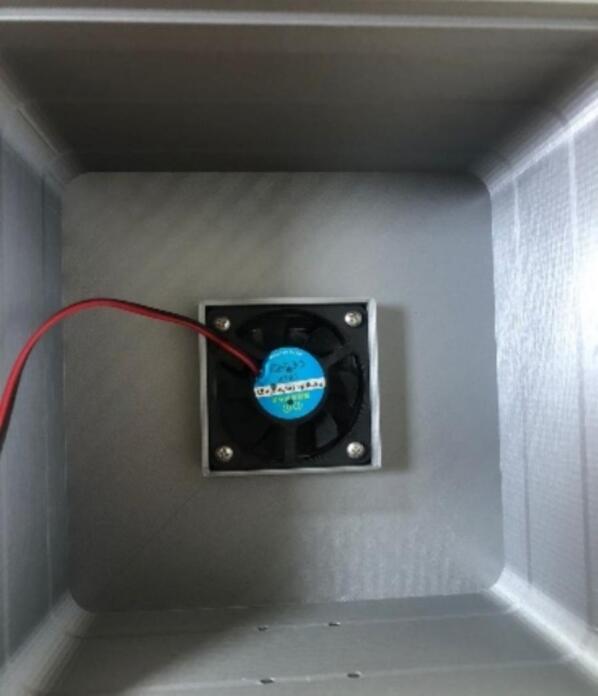
Securing fan and plate.
• Secure supports with the top and lower parts of the protective case using 4 × 5/8 screws, as shown in Fig. 24.

**Fig.24:**
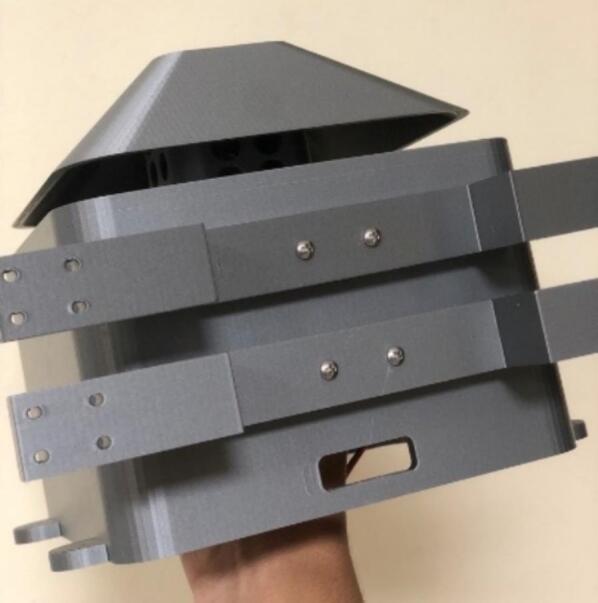
Securing the supports.
• The cable protectors are inserted into the holes in the lower part of the protective case. This allows the insertion of communication cables to acquire data from the monitoring device. See Fig. 25.

**Fig.25:**
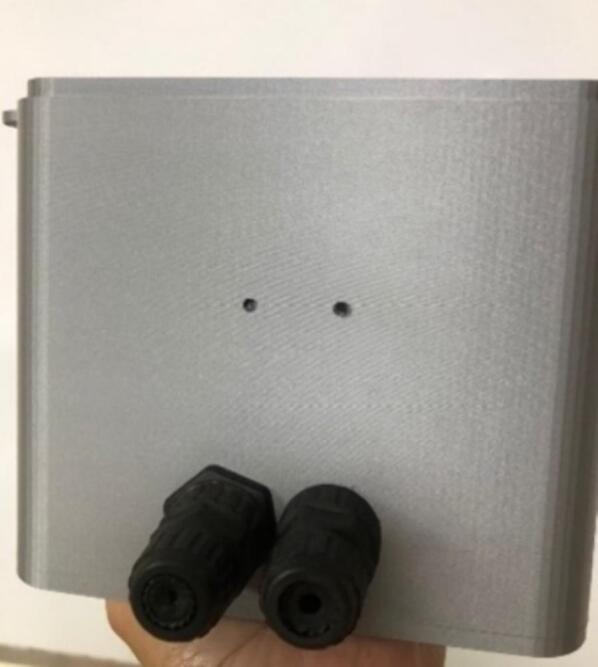
Insert the outputs.
• The monitoring device was installed inside the case. Communication and power cables were inserted through cable outlet holes and connected to the monitoring system. The fan was connected to the battery.
• To close this protective case, both parts were assembled using notches and secured using 4 × 3/8 screws, as shown in Fig. 26. Suitable screws and dowels must be used to mount and secure the monitoring system, depending on the surface material.

**Fig.26:**
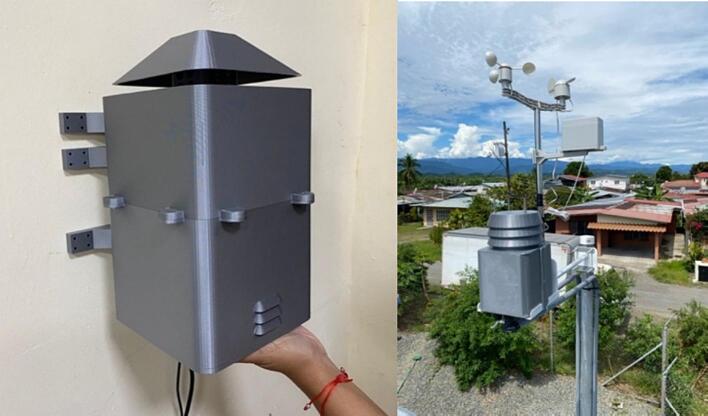
Assembled and deployed case.
• It is important to apply a silicone sealant to each slot, entry hole, and bolt hole to prevent the entry of water and dust. The area was cleaned and dried prior to the application of the silicone.

## 7 Operation instructions

### 7.1 Sensor calibration

The analog readings from the sensors allow us to determine the concentration of the gas, which is commonly measured in parts per million (ppm), parts per billion (ppb), or micrograms per cubic meter (µg/m^3^).

Before initiating the calibration procedure for the sensors, it is crucial to undergo a preheating phase. This entails keeping the sensor connected for a designated duration until stable measurements are achieved. The preheating period spans from 12 to 24 h, contingent upon the specific sensor.

In executing the calibration process, careful attention must be given to the datasheets accompanying the sensors. These documents provide graphical representations facilitating the derivation of gas concentration based on the correlation between sensor resistance (R0) and measured resistance (RS). Additionally, it is imperative to ascertain the load resistance (RL) employed in the module for reading the MQ sensor. Gas concentration is calculated according to:

(1) $$\mathrm{pollutionconcentrarion}=10^{Alog\left(\frac{\mathrm{Rs}}{R0}\right)+B}$$

In this equation, *RS* is the sensor resistance in ohms at the measured concentration that changes depending on the concentration of gas, *R0* is the sensor resistance in ohms at a known concentration without the presence of other gases, and *A* and *B* are calibration constants. The calibration line is derived by choosing two points P0(X0,Y0) and $P1\left(X1,Y1\right)$ on the graph. The line equation is given by:

(2) Y=Ax+B

where $A=\frac{Y1-Y0}{X1-X0}$ and B=Y0-AXo.

The output of MQ sensors is often provided in parts per million (ppm) or milligrams per cubic meter (mg/m^3^).

To calibrate and validate our sensors, we used the Aeroqual Series 500, a portable air pollution meter widely used in the environmental protection sector to measure the concentrations of pollutants. This equipment undergoes a rigorous procedure for calibration both in the factory and in the field. For both calibration procedures, suitably qualified laboratories are used to ensure the Aeroqual Series 500 works perfectly. The Aeroqual Series 500 used in this work has been field-calibrated every year in a certified laboratory to guarantee high accuracy and precision when measuring pollutants.

The sensors to be calibrated were placed alongside the Aeroqual Series 500 reference sensor in identical environmental conditions to ensure consistency. Simultaneous readings from both the sensors to be calibrated and the Aeroqual reference sensor were collected over a 14-day period at regular intervals. The readings from the sensors were compared with the Aeroqual sensor readings, and the Root Mean Square Error (RMSE) was calculated to quantify the performance of each sensor. The RMSE values after calibration were as follows: PM2.5: 0.5068, PM10: 2.0992, NO2: 0.0197, SO2: 0.0117, O3: 0.0213, and CO: 0.25.

If the RMSE values were higher than expected, indicating a discrepancy between the sensor readings and the reference sensor, the following steps were taken to calibrate the sensors:

• Offset Correction: An offset correction was applied to the sensor readings to remove any systematic bias.
• Gain Adjustment: Scaling factor adjustments were made to correct for discrepancies in sensor sensitivity.

These adjustments were made iteratively to minimize the RMSE, ensuring the sensor readings closely matched the reference sensor.

On the other hand, the metrological sensors were calibrated by comparing their readings with those from a standard meteorological station. The DHT22 sensor was in an area free from artificial heat sources to avoid bias due to the fan or other artifacts that could affect temperature readings. The comparison was conducted over the same 14-day period to ensure consistency in environmental conditions. The calibration involved the same steps as for the gas sensors, including offset correction and gain adjustment, to align the DHT22 readings with those from the standard meteorological station.

The calibration of the SEN-15901 Meteorological Station was conducted by addressing each of its components individually. For the rain gauge, known amounts of water were poured into the gauge, and the readings were compared with the expected values; adjustments were made in the software to ensure accuracy. For the anemometer, a controlled wind source was used to generate known wind speeds, which were then compared with readings from a reference anemometer. Calibration constants were adjusted to align the sensor readings with the reference values. The weathervane was calibrated by setting it to known directions using a protractor and comparing the readings. Adjustments were made to the calibration constants to ensure accurate directional readings. The SparkFun Weather Shield was utilized to read the data from these sensors and send it to the Arduino board, where the calibration adjustments were applied.

### 7.2 Communication network

The Arduino Yún stands as a microcontroller board seamlessly merging the capabilities of Linux with the user-friendly nature of Arduino. Featuring an ATmega32u4 microcontroller and a distinct Atheros AR9331 chip operating on a Linux distribution, it enables connectivity to the Internet through both Ethernet and Wi-Fi. Below is a fundamental guide illustrating the process of connecting the Arduino Yún to the Internet utilizing both Ethernet and Wi-Fi connections:

Hardware Requirements: Arduino Yún board, USB cable for programming, Ethernet cable, Wifi network, and Computer with the Arduino IDE installed.

Ethernet connection

a) Hardware Setup:
• Connect one end of the Ethernet cable to the Ethernet port on the Arduino YUN.
• Connect the other end of the Ethernet cable to the router or network switch.
• Power up the Arduino YUN using a USB cable connected to a power source or a computer.
b) Access to the YUN Configuration Page:
• Open a web browser and type the following address: https://arduino.local or https://192.168.240.1. This will give access to the YUN configuration page.
c) Configuration of network settings:
• Once the configuration page is accessed, navigate to the “Configuration” section.
• Enter the necessary network settings such as the IP address, subnet mask, gateway, and DNS servers. You can typically set these to be automatically obtained via DHCP or enter them manually if you have specific requirements.
d) Test Connection:
• Save the changes and reboot the Arduino YUN.
• After the reboot, check whether the Arduino YUN is connected to the Internet by running a simple test sketch that involves making an HTTP request or uploading a sample sketch that uses the Ethernet library.
e) Use Arduino IDE:
• If you are using the Arduino IDE, ensure that it is configured to communicate with the Arduino YUN over the network. In the Arduino IDE, go to “Tools” > “Port” and select the network port that corresponds to your Arduino YUN.
f) Upload and Run Sketches:
• You can now upload and run Arduino sketches on your Arduino YUN just like you would with any other Arduino board.
• Keep in mind that these steps provide a general guide, and the exact steps may vary depending on the specific firmware version of the Arduino YUN. Always refer to the official documentation and resources provided by Arduino for the most accurate and updated information.

WiFi connection

a) Hardware Setup:
• Connect the Arduino YUN to the computer using a USB cable.
• Ensure that the YUN is powered up.
b) Access to the YUN Configuration Page:
• Open a web browser and type the following address: https://arduino.local or https://192.168.240.1. This will take you to the YUN’s configuration page.
c) Configure Wi-Fi (Optional):
• If Wi-Fi is used, click the “Configure” button under the “Wi-Fi” section on the configuration page.
• Enter the details for your Wi-Fi network and click “Configure & Restart.”
d) Set Up Bridge Configuration:
• On the configuration page, go to the “Configuration” tab.
• Under the “Bridge” section, set the “Bridge Control” to “Serial”.
• Set the “Console” to the Serial Port (e.g., /dev/ttyATH0).
e) Upload a Sketch:
• Open the Arduino IDE on a computer.
• In the Arduino IDE, select the appropriate board and port from the “Tools” menu.
• Write or open a sketch that requires internet connectivity.
• Click the “Upload” button in the Arduino IDE to upload the sketch to Arduino YUN.
f) Monitoring the Serial Output:
• Open the Serial Monitor in the Arduino IDE to view the output of the sketch.
• This will help to debug and check if the YUN is successfully connected to the Internet.
g) Use Bridge Library:
• When writing a sketch, the bridge library can be used to communicate between the microcontroller and Linux side of the YUN.

**Fig.27:**
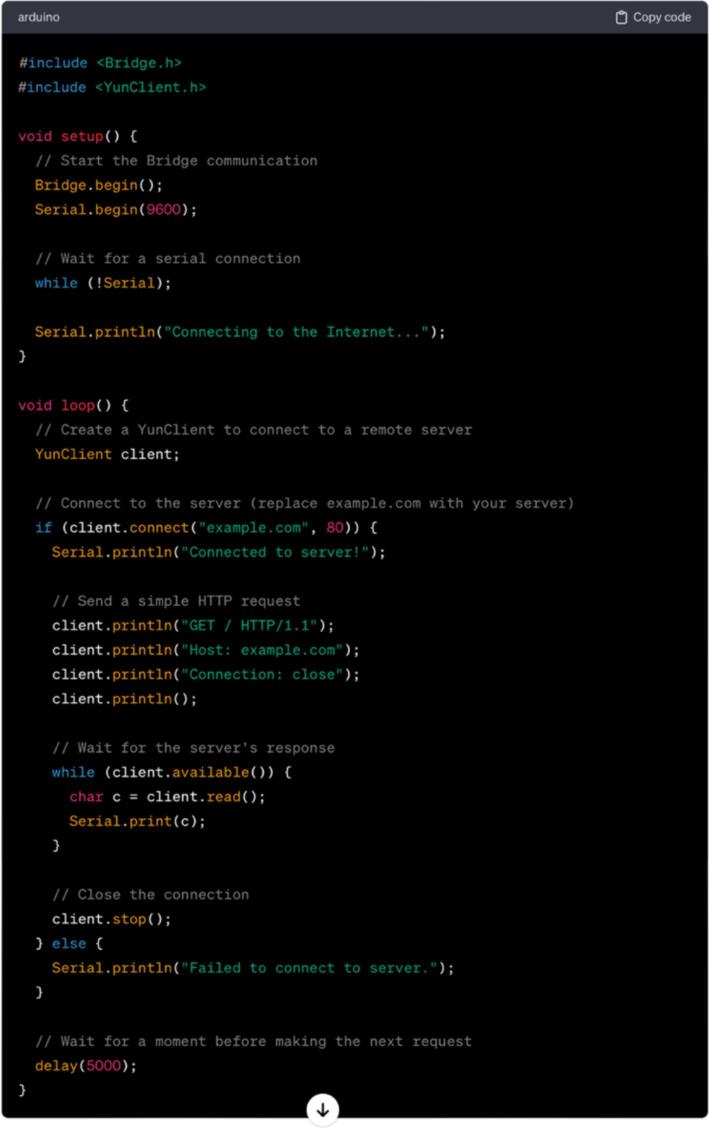
Example code as a starting point.

In Fig. 27, a straightforward example sketch is depicted, showcasing the connectivity to the Internet through the YUN’s Linux processor.

Once these steps have been completed, the monitoring station is ready to send data to the data-visualization platform.

### 7.3 Data visualization platform

**Fig.28:**
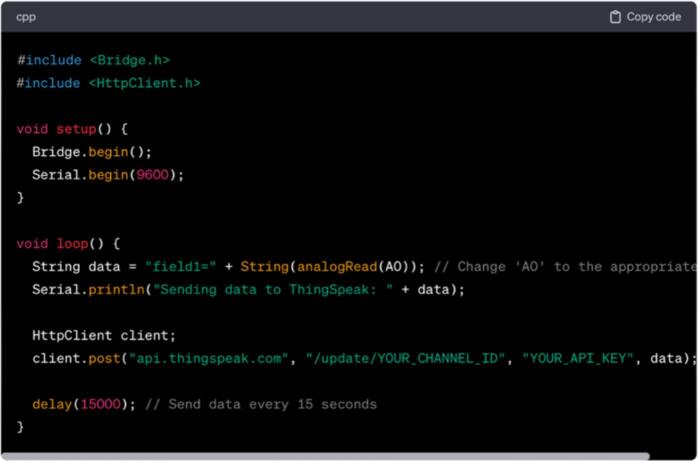
Example code as a starting point.

Connecting the monitoring station to ThingSpeak involves several steps (Fig. 28). Here, a step-by-step guide on how to connect Arduino YUN within the station to ThingSpeak is as follows:

a) Set Up a ThingSpeak Account:
• Go to the ThingSpeak website (https://thingspeak.com/).
• Once registered, log into the ThingSpeak account.
b) Create a Channel:
• After logging in, click on “Channels” in the top menu.
• Click on “New Channel” to create a new channel.
• Fill in the necessary information, such as Name, Field labels, among others.
• Click on “Save Channel” to create the channel.
c) Get Your Write API Key:
• On your ThingSpeak channel, click on the “API Keys” tab.
• Find the “Write API Key” and save it. This key is used by Arduino to send data to ThingSpeak.
d) Set Up Arduino YUN:
• Connect your Arduino YUN to your computer via USB.
e) Install Required Libraries:
• Open the Arduino IDE on a computer.
• Go to “Sketch” −> “Include Library” −> “Manage Libraries.”
• Search for “Bridge” and install a bridge library.
f) Write Arduino Code:
• Open a new sketch in the Arduino IDE.
• Replace “YOUR_CHANNEL_ID” and “YOUR_API_KEY” with the ThingSpeak channel ID and write the API key.
• Adjust the data sent to ThingSpeak according to your requirement.
g) Upload the Code:
• Upload the code to the Arduino YUN.
h) Monitor Serial Output:
• The Serial Monitor in the Arduino IDE is opened to see the debug information.
i) Check ThingSpeak Channel:
• Go to the ThingSpeak channel and check if the data are updated.

### 7.4 Deployment and maintenance

Establishing air pollution monitoring station for accurate measurements demands thoughtful consideration of multiple factors. The ideal placement of the is contingent upon the distinct objectives of the monitoring program, the prevalent pollution sources in the region, and the properties of the pollutants under scrutiny. Table 5 outlines overarching guidelines to aid in the selection of suitable location for air pollution monitoring station.

**Table 5:** General guidelines for selecting locations for air pollution monitoring stations.

Representative Locations	Place monitoring stations in areas that are representative of the overall air quality in the region. Consider factors such as land use, traffic patterns, industrial activities, and population density.
Proximity to Pollution Sources	Install stations near major sources of pollution, such as industrial facilities, traffic corridors, and areas with high combustion activities.
Background Sites	Include background sites that are away from direct pollution sources to measure baseline or background levels of pollutants.
Urban and Rural Areas	Place monitoring stations in both urban and rural areas to capture the differences in pollution levels between these environments. Urban areas often have higher concentrations due to increased human activities.
Elevation Differences	Consider placing stations at different elevations, as air quality can vary with altitude. This is particularly important in regions with significant topographical variations. In this system, the monitoring station were placed at 5 m above ground.
Meteorological Considerations	Consider meteorological factors, such as prevailing wind patterns. Monitoring stations should be strategically located to capture the impact of air masses carrying pollutants into the region. This system is equipped with a meteorological station to satisfy this requirement.
Community Exposure	Install stations in areas where people live, work, and spend significant amounts of time.
Regulatory Compliance	Adhere to regulatory requirements and guidelines for station placement, especially if the monitoring is conducted for compliance purposes. The proposed system was deployed following standards and recommendations from the Environmental Protection Agency (EPA) – USA.
Data Integration	Integrate data from multiple monitoring stations to create a comprehensive picture of air quality across the region.
Accessibility and Safety	Ensure that monitoring stations are easily accessible for maintenance and data retrieval. Safety considerations are crucial, especially in industrial areas or locations with potential hazards.
Public Health Concerns	Consider public health concerns and place monitoring stations in areas with known health risks or where vulnerable populations are present.

In essence, a well-crafted air quality monitoring network considers the distinctive attributes of the region and the targeted pollutants. The synergy among environmental scientists, meteorologists, and relevant authorities is crucial in devising an efficient monitoring strategy.

Routine maintenance activities are conducted to ensure the system operates optimally. These tasks encompass cleaning the station and sensors, assessing the condition of station protection cases, conducting on-site data validation tests, executing connectivity tests, and performing access tests on the platform and database, among other responsibilities. The scheduling of maintenance tasks is tailored to the site’s specific characteristics where the station is deployed. In Panama, for instance, this maintenance work is carried out monthly, aligning with the country’s tropical climatic conditions.

### 7.5 Air pollution monitoring system

Following the assembly of the station according to the previously provided instructions, the system is ready for deployment. The process of installing and activating the proposed air pollution monitoring system encompasses several steps. While the subsequent guide offers a general overview, it’s important to acknowledge that specific instructions may differ based on the monitoring objectives and local regulations. First, it is important to define the objectives and requirements of the study. In this step, users must identify the specific pollutants they want to monitor, define the geographical area, and scope of monitoring, and understand the regulatory compliance to ensure that the monitoring system complies with local and international air quality regulations. Users must then decide the monitoring location and analyze its requirements. Table 4 summarizes some of the requirements for selecting locations for the air pollution monitoring station. Based on the location, some technical characteristics are selected for the monitoring station. Users can choose the appropriate sensors based on the pollutants under the study, evaluate factors such as accuracy, sensitivity, and calibration requirements, determine communication protocols Wi-Fi or Ethernet (the proposed station have both communication protocols), and decide on a data logging method (local storage or cloud-based platform). Once the system is installed, users can select their own monitoring and data management capabilities, such as setting up the sampling period, configuring alerts for threshold exceedances, choosing visualization tools to interpret data trends (tabular or graphical), and implementing tools for analyzing the collected data. The system is compatible with any IoT data visualization platform that can be configured to receive data from IoT devices, allowing users to select the platform that best suits their preferences. Finally, users must establish quality control by regularly evaluating and calibrating sensors, reviewing monitoring results, adjusting monitoring locations or parameters if necessary, establishing a routine maintenance schedule for sensor cleaning and system checks, developing regular reports for internal use and compliance reporting, ensuring that the collected data meet regulatory reporting requirements, and guaranteeing data is easily accessible to other users.

By following these steps and consulting experts in air quality monitoring, it is possible to establish a robust air pollution monitoring system.

## 8 Validation and characterization

### 8.1 Evaluation of the air pollution sensors calibration

In this section, we evaluate the calibration of the air pollution sensors by comparing the acquired measurements with those obtained from the Aeroqual Series 500. The comparison revealed close alignment between the values obtained from both devices, with a minimal measurement error. This observation suggests accurate calibration of the air pollution sensors, affirming the precision and reliability of the obtained values. The depicted graphs were generated by collecting data from both the sensors and the Aeroqual Series 500 over a continuous measurement period of approximately 15 days, with samples taken at 5-minute intervals.

**Fig.29:**
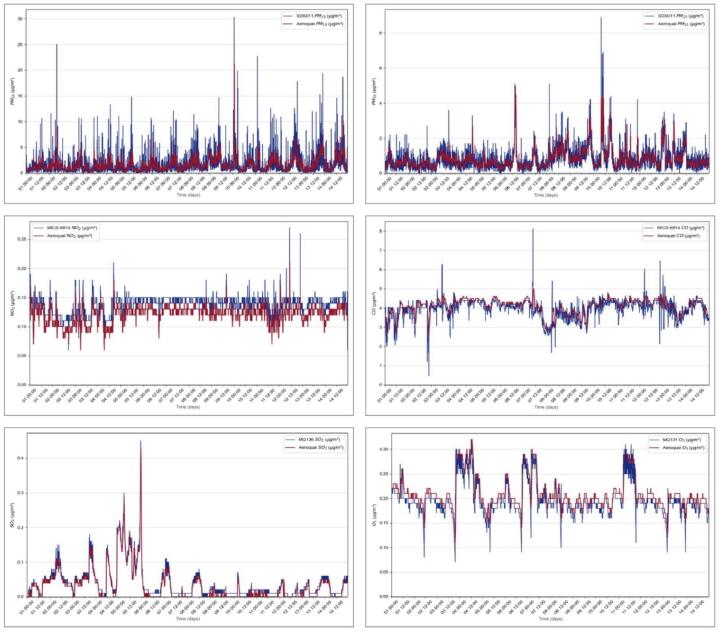
Comparison between air pollution sensors and Aeroqual Series 500.

The calibration data presented in Fig. 29 illustrates the performance of our sensors over a 14-day period. The PM2.5 and PM10 sensors show a high degree of correlation with the Aeroqual reference sensors, with RMSE values of 0.5068 and 2.0992 respectively, indicating consistent performance in measuring particulate matter. The NO2 sensor also demonstrates reliable performance with an RMSE of 0.0197, closely tracking the Aeroqual NO2 sensor readings. Similarly, the SO2 sensor exhibits strong agreement with the reference sensor, reflected in an RMSE of 0.0117, confirming its reliability in detecting sulfur dioxide levels. The O3 sensor maintains accurate readings compared to the Aeroqual O3 sensor, with an RMSE of 0.0213, demonstrating its effectiveness in measuring ozone concentrations. Lastly, the CO sensor shows excellent stability and accuracy with an RMSE of 0.25, closely following the Aeroqual CO sensor’s measurements. These results, observed under varying environmental conditions over an extended calibration period, validate the robustness and reliability of our air quality monitoring system.

### 8.2 Validation of air pollution monitoring system

**Fig.30:**
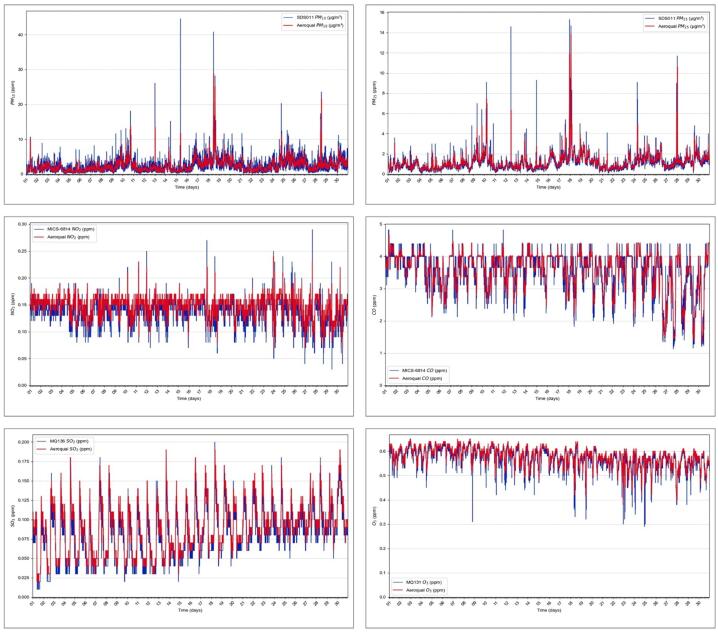
Validation of air pollution monitoring system.

Following the calibration of the sensors, the monitoring station was strategically positioned in a location, following the guidelines on Table 5, to examine the patterns of air pollutants. Fig. 30 illustrates the outcomes of monitoring various air pollutants, including particulate matter (PM_2.5_ and PM_10_), nitrogen dioxide (N_O2_), sulfur dioxide (SO_2_), carbon monoxide (CO), and ozone (O_3_). These graphs depict data collected from the sensors over an extended continuous measurement period of 30 days, with samples taken every 5 min.

To ensure the accuracy and reliability of our measurements, we installed the monitoring system next to an Aeroqual Series 500 for the entire validation period. This comparison allowed us to confirm that the sensors maintained their calibration without significant drift, validating the reported results. The close alignment between the sensor data and the Aeroqual Series 500 data, with minimal measurement error, supports the reliability of our sensors. Additionally, the field deployment of the sensors was meticulously planned and executed. The deployment locations were chosen to avoid artificial heat sources and other potential interferences.

The observed capability of the sensors to provide real-time measurements of air pollution underscores the successful design and calibration of the station. This accomplishment is crucial in system development, ensuring the deployment of measurement stations capable of real-time and accurate monitoring. Beyond real-time monitoring, the collected data offers insights into the behavior of each variable, enabling users to identify patterns, comprehend pollution impacts, predict future trends, and extract valuable information such as maximum, minimum, and average values, as well as generate meaningful indicators.

### 8.3 Validation of protective case

Ensuring the proper operation of monitoring stations necessitates the creation of a protective case designed to sustain a consistent and suitable environment internally. This environment is crucial for the optimal functioning of electronic components such as microcontrollers, sensors, modules, and other essential elements. Table 6 shows the recommended values of temperature and relative humidity for the main electronics components of the stations.

**Table 6:** Temperature and relative humidity requirements.

Electronic component	Temperature (${\;}^{{}^{\circ}}$ C)	Relative humidity (%)
CJMCU MICS 6814	−30.0–85.0	5–95
SDS 011	−10.0–50.0	Less than 90 %
MQ 136	−20.0–70.0	Less than 95 %
CJMCU MQ 131	−10.0–50.0	Less than 95 %
Arduino YUN	−40 °C to 85 °C	30 % to 90 %
Arduino NANO	−40 °C to 85 °C	30 % to 90 %

The proposed protective case was validated using SolidWorks Flow Simulation, which is a Computational Fluid Dynamics (CFD) software. Mixing computational fluid dynamics (CFD) software with a computer-aided design (CAD) system allows the use of simulation and design stages simultaneously to evaluate alternatives. SolidWorks is an intuitive tool that allows us to vary conditions and observe behavior within the proposed models.

**Fig.31:**
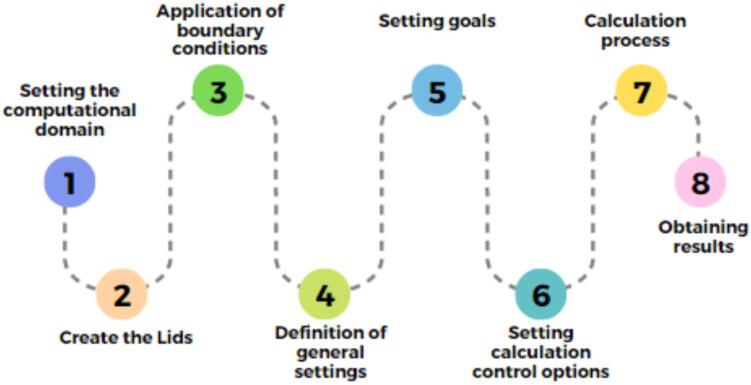
Steps to validate the proposed models.

To confirm the validity of the proposed models, it is essential to have a comprehensive design for each component. Subsequently, we can move forward with simulations, adhering to the outlined steps in the diagram presented in Fig. 31. These simulations account for three distinct wind speeds, encompassing various scenarios observed during the data collection period. The considered wind speeds are 11.8, 13.1, and 14.4 m/s, determined by averaging the speeds generated by the fans employed in the developed system.

**Fig.32:**
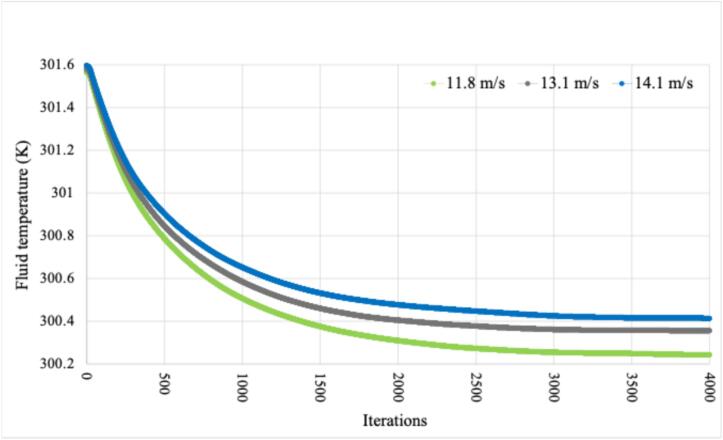
Average Fluid Temperature − PLA.

In Fig. 32, the average fluid temperature is presented. It can be observed that the lower the wind speed the lower the average fluid temperature inside the device. However, the variation in the average fluid temperature is very small and can be neglected. After some simulation iterations, the average fluid temperature converges to 300.2–300.4 K, corresponding approximately to 26–27 °C. This result shows that the proposed protective case no only protects the internal electronic devices from environmental factors, but also guarantees to have a fluid temperature within the required operation values presented in Table 5, regardless of the speed or type of fan selected.

**Fig.33:**
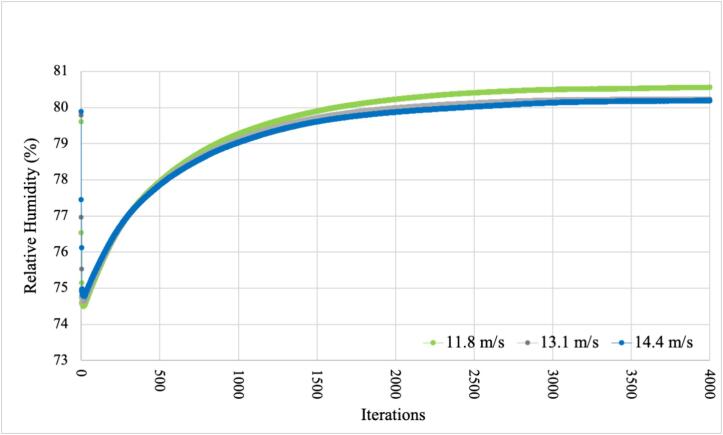
Average Relative Humidity – PLA.

The behavior of the average relative humidity inside the monitoring station is presented in Fig. 33. In this case, it is observed that the lower fan speeds the higher the average relative humidity inside the device. However, there are no notable changes in the general behavior of the relative humidity of the wind speed changes. Therefore, regardless of the speed or type of fan selected, the protective case maintains the relative humidity inside the device within desired values, while protecting the internal devices from dangerous factors. Notice that after some iterations, the average relative humidity converges to 80–80.5 %, satisfying the values required in Table 5 for all de main devices implemented in the proposed system.

It can be said that the proposed design for the protective case satisfies the operating requirements for both temperature and relative humidity, guaranteeing optimal operation conditions to the main electronic components in the air pollution monitoring system.

## 9 Conclusion

The proposed system provides the environmental protection sector with an efficient and easy-to-use technological solution that aims to monitor air pollutants by applying an ICT-based system to remotely monitor air pollution variables that affect quality of life of living beings. Specifically, this work proposes a low-cost Internet of Things (IoT)-based air pollution monitoring system with protective case that consists of a single station connected to an IoT platform through a communication network. Each station has multiple sensors to measure ambient temperature and relative humidity, concentrations of carbon monoxide (CO), nitrogen dioxide (NO_2_), sulfur dioxide (SO_2_), suspended particles (PM_2.5_ and PM_10_), and ozone (O_3_). The measurements obtained from the sensors are sent to a server through an Ethernet network. Finally, the information in the server is analyzed by the users on a data visualization platform that present results in real time either in tables or graphs. With this system, any authorized user can access to the platform from any electronic device capable of connecting to the Internet.

To develop the system, we employed materials, equipment, and energy-efficient electronic devices for electrical supply, monitoring, data processing, communication, and visualization. Designing PCB circuits using CNC machines and creating protective cases with 3D printers were essential for each monitoring station, especially for tropical climate environments where this system was deployed. Additionally, electronic support and specialized interconnections were implemented based on the electronic system design. The overall cost of designing this station was relatively low compared to commercial monitoring systems, enabling a variety of users to adopt this technology. The station was specifically designed to ensure low energy consumption, making them suitable for deployment in remote locations. The development process also involved designing algorithms for the monitoring station, communication modules, and the IoT platform. Implementation required installing each monitoring station, validating the measurements obtained, and analyzing the network’s performance.

The study’s findings revealed the monitoring system’s efficient real-time measurement of air pollution variables, furnishing crucial insights into factors impacting crop production within greenhouses. Results indicated distinct behavioral patterns in most of the studied variables, facilitating the early detection of critical situations to preempt undesired situations. This proactive approach ensures optimal conditions and minimizes impact on health caused by air pollutants. This information not only empowers citizen and entities to comprehend the behavior of air pollutants but also enables researchers to formulate indicators to address air pollution and climate change’s potential impact on the environmental protection sector.

The adoption of an ICT-based remote monitoring solution minimizes measurement time and decreases manual measurement errors. This implementation aims to assist both public and private research institutions in studying air pollution behavior under specific weather conditions. A future extension involves integrating an expert system, utilizing artificial intelligence (AI) technologies, to interpret collected data, identify issues, and propose solutions.

## CRediT authorship contribution statement

**Edwin Collado:** Writing – review & editing, Writing – original draft, Supervision, Investigation, Funding acquisition. **Sallelis Calderón:** Writing – original draft, Software, Methodology, Investigation, Conceptualization. **Betzaida Cedeño:** Validation, Software, Methodology, Conceptualization. **Olga De León:** Software, Methodology, Conceptualization. **Miriam Centella:** Validation, Software, Methodology, Conceptualization. **Antony García:** Writing – review & editing, Validation. **Yessica Sáez:** Writing – review & editing, Supervision, Funding acquisition.

## Declaration of competing interest

The authors declare the following financial interests/personal relationships which may be considered as potential competing interests: Edwin Collado reports financial support was provided by National Secretariat for Science Technology and Innovation. Yessica Saez reports financial support was provided by National Researcher System. If there are other authors, they declare that they have no known competing financial interests or personal relationships that could have appeared to influence the work reported in this paper.
